# Supplementary material for: Autoregulation of the MET receptor tyrosine kinase by its intracellular juxtamembrane domain
Source: Biochem J. 2025 Dec 17;482(24):1859–75. doi: 10.1042/BCJ20253378 (PMC12751062; doi:10.1042/BCJ20253378)
Supplement: 1undefined [file bcj-482-24-BCJ20253378-s001.pdf]

Supplementary Data 1

Linossi et al

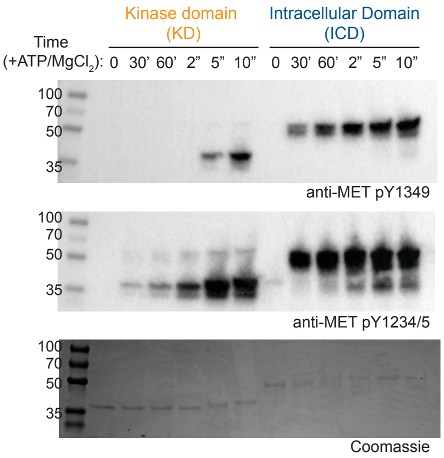

Experiment A
(Figure 1D)

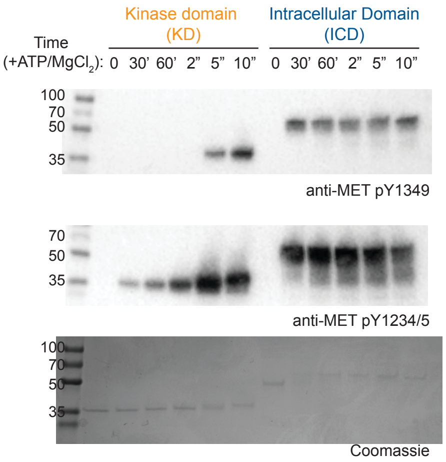

Experiment B

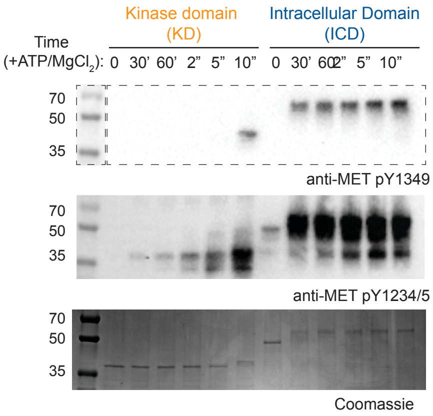

Experiment C

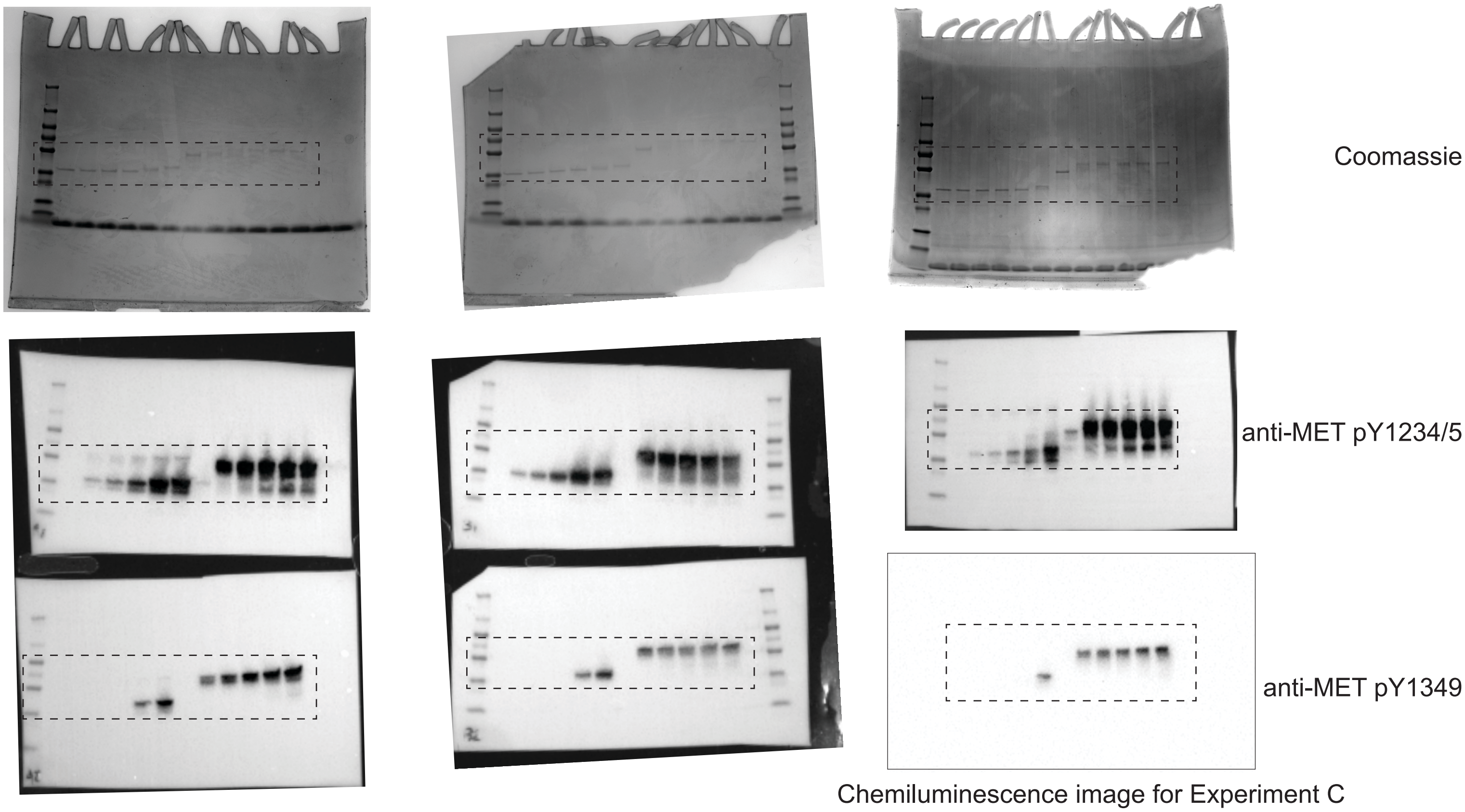

Chemiluminescence image for Experiment C
Boxed area represents region used for Experiment C image
See image below for blot dimensions and edges

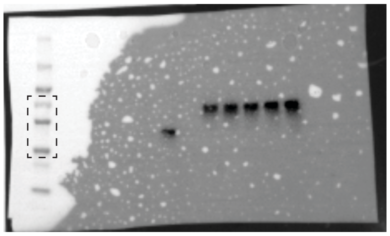

Colorimetric image for molecular weight ladder
overlay for Experiment C
Boxed area represents region used for Experiment C image
