# Supplementary figures and images for: Autoregulation of the MET receptor tyrosine kinase by its intracellular juxtamembrane domain
Source: Biochem J. 2025 Dec 17;482(24):1859–75. doi: 10.1042/BCJ20253378 (PMC12751062; doi:10.1042/BCJ20253378)

# Supplementary Figure 3

Linossi et al

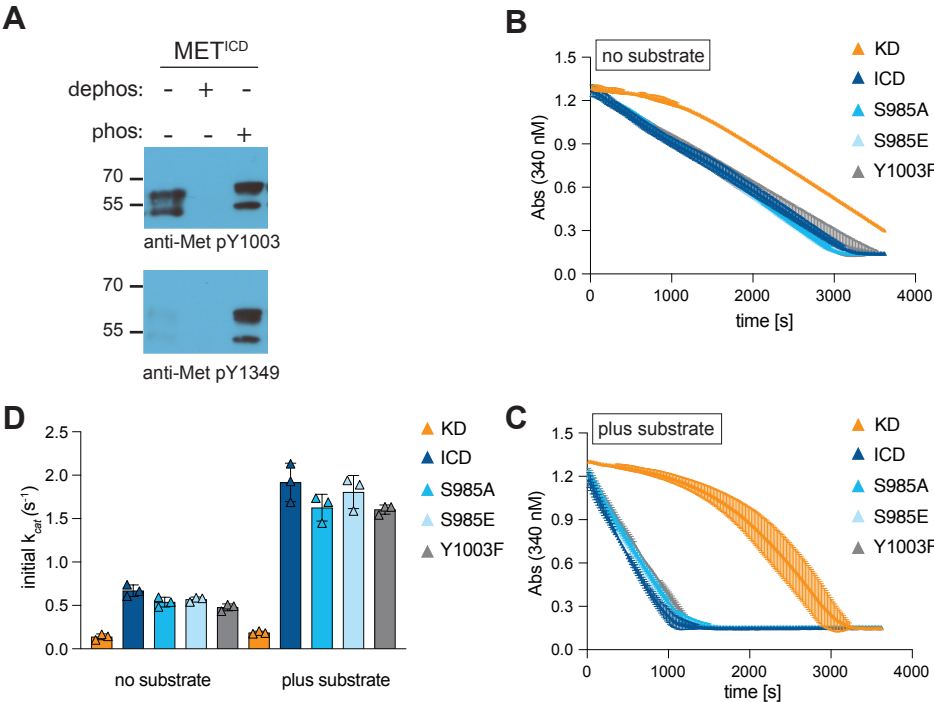

Supplement: online supplementary figure 3. [file bcj-482-24-BCJ20253378-s002.pdf]

# Supplementary Figure 1

Linossi et al

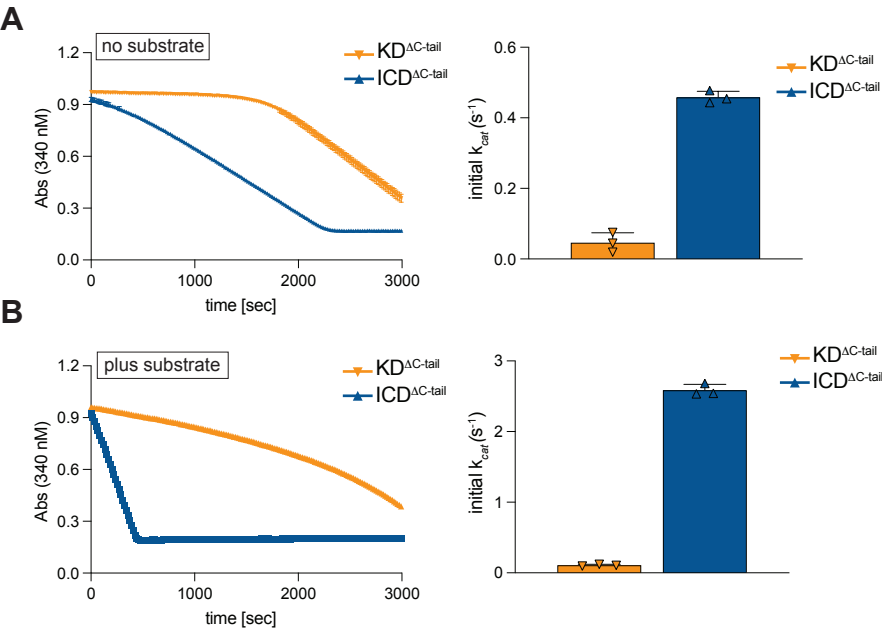

Supplement: online supplementary figure 1. [file bcj-482-24-BCJ20253378-s006.pdf]

# Supplementary Figure 2

Linossi et al

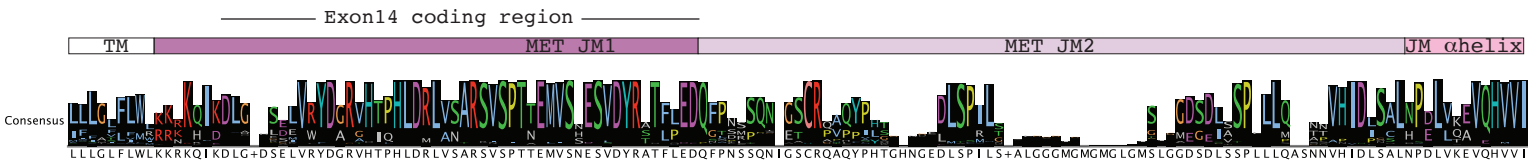

Supplement: online supplementary figure 2. [file bcj-482-24-BCJ20253378-s007.pdf]
