# Supplementary material for: Autoregulation of the MET receptor tyrosine kinase by its intracellular juxtamembrane domain
Source: Biochem J. 2025 Dec 17;482(24):1859–75. doi: 10.1042/BCJ20253378 (PMC12751062; doi:10.1042/BCJ20253378)
Supplement: online supplementary table 1. [file bcj-482-24-BCJ20253378-s003.pdf]

| Protein | Autophosphorylation $k_{cat}$ ($S^{-1}$) ± SEM | n= | p (vs KD) | p (vs ICD) | Substrate dependent $k_{cat}$ ($S^{-1}$) ± SEM ± SEM | n= | p (vs KD) | p (vs ICD) |
|---|---|---|---|---|---|---|---|---|
| KD | 0.104 ± 0.023 | 9 | - | <0.0001 | 0.168 ± 0.019 | 9 | - | <0.0001 |
| ICD | 0.466 ± 0.036 | 9 | <0.0001 | - | 1.546 ± 0.172 | 9 | <0.0001 | - |
| $KD^{\Delta C\text{-tail}}$ | 0.052 ± 0.017 | 3 | ns | - | 0.167 ± 0.033 | 3 | ns | - |
| $ICD^{\Delta C\text{-tail}}$ | 0.394 ± 0.091 | 2 | - | ns | 2.707 ± 0.122 | 2 | - | 0.0015 |
| $ICD^{\Delta JM1}$ | 0.055 ± 0.013 | 5 | ns | <0.0001 | 0.131 ± 0.030 | 3 | ns | <0.0001 |
| $ICD^{\Delta JM2}$ | 0.268 ± 0.065 | 3 | ns | ns | 2.021 ± 0.088 | 2 | 0.0234 | 0.0381 |
| $ICD^{JM2=GSL}$ | 0.680 ± 0.079 | 2 | ns | ns | 1.833 ± 1.074 | 2 | ns | ns |
| $ICD^{Y1003F}$ | 0.433 ± 0.016 | 4 | <0.0001 | ns | 1.466 ± 0.092 | 4 | 0.0005 | ns |
| $ICD^{S985A}$ | 0.449 ± 0.038 | 5 | <0.0001 | ns | 1.407 ± 0.231 | 5 | 0.0057 | ns |
| $ICD^{S985E}$ | 0.470 ± 0.058 | 3 | 0.0137 | ns | 1.571 ± 0.153 | 3 | 0.0107 | ns |
| TPR-MET | 0.147 ± 0.025 | 2 | ns | 0.0163 | 0.294 ± 0.082 | 2 | ns | 0.0016 |
| $TPR\text{-}MET^{+Ex14}$ | 0.267 ± 0.002 | 2 | ns | ns | 0.729 ± 0.143 | 2 | 0.0459 | 0.0219 |
| KD (phos) | 0.944 ± 0.440 | 3 | - | ns# | 3.915 ± 0.825 | 3 | - | ns# |
| ICD (phos) | 1.003 ± 0.366 | 3 | ns# | - | 2.895 ± 0.801 | 3 | ns# | - |
| $RON^{KD}$ | 0.036 ± 0.004 | 3 | - | 0.0052## | 0.076 ± 0.011 | 3 | - | ns |
| $RON^{ICD}$ | 0.069 ± 0.002 | 3 | 0.0052## | - | 0.072 ± 0.006 | 3 | ns | - |
| $RON^{\Delta JMB}$ | 0.086 ± 0.018 | 3 | ns | ns | 0.099 ± 0.007 | 3 | ns | 0.0452## |
| $RON^{EDE\text{-}AAA}$ | 0.083 ± 0.010 | 3 | 0.0278## | ns | 0.145 ± 0.036 | 3 | ns | ns |

**Supplementary Table 1. Summary values and statistical analysis from coupled kinase assays.** Average values from independent experimental means and SEM values are shown. Each independent experiment was conducted with at least three technical replicates. p-values were calculated using Welch's t-test on independent experimental means. The autophosphorylation or substrate dependent activity of the MET KD and ICD were compared to all other dephosphorylated MET and TPR-MET constructs. #Equivalent statistical tests were performed comparing the activity of the phosphorylated KD and ICD to each other. ##The activity of the RON KD and ICD were compared to each other and the RON ICD mutants. SEM = standard error of the mean; ns = not significant.
