# Supplementary material for: Autoregulation of the MET receptor tyrosine kinase by its intracellular juxtamembrane domain
Source: Biochem J. 2025 Dec 17;482(24):1859–75. doi: 10.1042/BCJ20253378 (PMC12751062; doi:10.1042/BCJ20253378)
Supplement: online supplementary table 2. [file bcj-482-24-BCJ20253378-s004.pdf]

Protein	Autophosphorylation RLU fold-change relative to KD ± SEM	n=	Substrate dependent RLU fold-change relative to KD ± SEM	n=
KD	1.00 ± 0.00	2	1.11 ± 0.11	2
ICD	2.82 ± 0.00	2	2.94 ± 0.54	2
TPR-MET	1.86 ± 0.30	2	1.23 ± 0.50	2
TPR-MET^{+Ex14}	2.47 ± 0.58	2	2.56 ± 0.62	2

Supplementary Table 2. Summary values of ADP-Glo assays. Average values from independent experimental means and SEM values are shown from ADP-Glo assays. Each independent experiment was conducted with at least four technical replicates. SEM = standard error of the mean; RLU = relative luciferase
