# Supplementary material for: Autoregulation of the MET receptor tyrosine kinase by its intracellular juxtamembrane domain
Source: Biochem J. 2025 Dec 17;482(24):1859–75. doi: 10.1042/BCJ20253378 (PMC12751062; doi:10.1042/BCJ20253378)
Supplement: online supplementary table 3. [file bcj-482-24-BCJ20253378-s005.pdf]

Supplementary Table 2. Details of recombinant protein constructs.

Protein	Construct boundaries	Description
KD	Q1048-S1390	Wildtype MET kinase domain
ICD	K956-S1390	Wildtype MET full intracellular domain
$KD^{\Delta C\text{-}tail}$	Q1048-G1346	Wildtype MET kinase domain lacking the C-terminal tail
$ICD^{\Delta C\text{-}tail}$	K956 - G1346	Wildtype MET full intracellular domain lacking the C-terminal tail
$ICD^{\Delta JM1}$	D1010-S1390	Wildtype MET intracellular domain lacking JM1 segment (K963-E1009)
$ICD^{\Delta JM2}$	K956-D1010-Q1048-S1390	Wildtype MET intracellular domain with deletion of JM2 segment (D1010-L1047)
$ICD^{JM2=GSL}$	K956-D1010-(GS)-Q1048-S1390	Wildtype MET intracellular domain with JM2 segment (D1010-L1047) replaced with an equivlanet length repeating GS linker of 37 amino acids
ICD^{Y1003F}	K956-S1390	Full MET intracellular domain with Y1003F mutation
ICD^{S985A}	K956-S1390	Full MET intracellular domain with S985A mutation
ICD^{S985E}	K956-S1390	Full MET intracellular domain with S985E mutation
TPR-MET	TPR(A2-T142)MET(D1010-S1390)	Oncogenic TPR-MET fusion protein
$TPR\text{-}MET^{+Ex14}$	TPR(A2-T142) MET(D963-S1390)	Oncogenic TPR-MET fusion protein with addition of D963-E1009 of the MET intracellular domain corresponding the exon 14 coding region
RON^{KD}	R1053-T1400	Wildtype RON kinase domain
RON^{ICD}	R983-T1400	Wildtype RON full intracellular domain
$RON^{\Delta JMB}$	R983-del(P1009-V1035)-T1400	RON intracellular domain lacking P1009-V1035
$RON^{EDE\text{-}AAA}$	R983-T1400	RON intracellular domain with E1044, D1045, E1046 mutated to alanines
